# Supplementary material for: Content-rich biological network constructed by mining PubMed abstracts
Source: BMC Bioinformatics. 2004 Oct 8;5:147. doi: 10.1186/1471-2105-5-147 (PMC528731; doi:10.1186/1471-2105-5-147)
Supplement: Additional File 2 — The original results of the above study (non-essential files are deleted to keep the file size under the limit set by BMC bioinformatics). [file 1471-2105-5-147-S2.bz2 › chilibotAdditionalFile2/dip10/264ID7597098E1063/html/CDC42_PAK1.html]

 


 **CDC42** and **PAK1** 
  
Found 308 abstracts in PubMed, retrieved 10.  
 

 What does Google say? 
 PDF only 
| .edu only 

---

**Interactive relationship** (e.g. stimulation, inhibition, etc)

**Neutral relationship**- Our results suggest that Gbetagamma binds  **PAK1**  and, via PAK  [ **PAK1** ]  associated PIXalpha, activates  **Cdc42** , which in turn activates  **PAK1** .  Ref: 12887923 Cell, 2003
- p21  [ **PAK1** ]  activated protein kinases PAK  [ **PAK1** ]  are a family of serine threonine protein kinases which are activated by binding of the p21  [ **PAK1** ]  G proteins  **Cdc42**  or Rac.  Ref: 12853446 J Biol Chem< MedlineTA>J Biol Chem, 2003
- The p21  [ **PAK1** ]  activated kinase PAK  [ **PAK1** ]  homolog, Shk1, is a critical component of a multi functional Ras  **Cdc42**  PAK  [ **PAK1** ]  complex required for viability, polarized growth and cell shape, and sexual differentiation in the fission yeast, Schizosaccharomyces pombe.  Ref: 12764130 J Biol Chem< MedlineTA>J Biol Chem, 2003
- Two reports in this issue show that activated  **Cdc42**  at the leading edge helps orient the cell s axis in a signaling complex with Gbetagamma,  **PAK1** , and PIXalpha.  Ref: 12887916 Cell, 2003
- The p21  [ **PAK1** ]  activated protein kinases Paks are serine threonine protein kinases activated by binding to Rho family small GTPases, Rac and  **Cdc42** .  Ref: 12860998 J Biol ChemJ Biol Chem, 2003
- Pak  [ **PAK1** ]  interacting exchange factor betaPix is a recently cloned protein that contains a multidomain with many potential binding sites and is known to be involved in the regulation of  **Cdc42**  Rac GTPases and Pak  [ **PAK1** ]  kinase activity.  Ref: 12691829 Cancer Lett, 2003
- **PAK1**  activity was abolished in cells expressing both  **Cdc42**  DN and Rac1 DN.  Ref: 12733988 Biochem J< MedlineTA>Biochem J, 2003

**Non-interactive relationship** (e.g. studied together, co-existance, homology, etc.)

- Differential Signaling by Muscarinic Receptors in Smooth Muscle m2 mediated inactivation of MLCK via Gi3,  **Cdc42**  Rac1, and  **PAK1** , and m3 mediated MLC20 phosphorylation via Rho kinase MYPT1 and PKC CPI 17 pathways.  Ref: 12733988 Biochem J< MedlineTA>Biochem J, 2003
- m2 receptors were coupled via Gbgi3 to activation of PLC b3, PI 3 kinase,  **Cdc42**  Rac1, and  **PAK1** , resulting in phosphorylation and inactivation of MLCK.  Ref: 12733988 Biochem J< MedlineTA>Biochem J, 2003
- We describe a signaling mechanism involving Gbetagamma, PAK  [ **PAK1** ]  associated guanine nucleotide exchange factor PIXalpha,  **Cdc42** , and p21  [ **PAK1** ]  activated kinase PAK 1  [ **PAK1** ] .  Ref: 12887923 Cell, 2003
- Thus, in this pathway,  **PAK1**  is not only an effector for  **Cdc42** , but it also functions as a scaffold protein required for  **Cdc42**  activation.  Ref: 12887923 Cell, 2003
- Mutation of histidine residues 19 and 22 to leucine on the p21  [ **PAK1** ]  binding domain of Pak5 completely abolished the binding of  **Cdc42**  and the  **Cdc42**  mediated autophosphorylation.  Ref: 12860998 J Biol ChemJ Biol Chem, 2003
- Taken together, these results suggest that Pak5, like  **Pak1** , contains an autoinhibitory domain and its activity is regulated by  **Cdc42** .  Ref: 12860998 J Biol ChemJ Biol Chem, 2003
- This Gbetagamma  **PAK1**  PIXalpha  **Cdc42**  pathway is essential for the localization of F actin formation to the leading edge, the exclusion of PTEN from the leading edge, directional sensing, and the persistent directional migration of chemotactic leukocytes.  Ref: 12887923 Cell, 2003
- MLCK phosphorylation was inhibited by  **PAK1**  antibody and in cells expressing  **Cdc42**  DN and Rac1 DN. m3 receptors were coupled via Gaq 11 to PLC b1 and via RhoA to activation of Rho kinase, PLD, and PKC.  Ref: 12733988 Biochem J< MedlineTA>Biochem J, 2003
- Directional Sensing Requires Gbetagamma Mediated  **PAK1**  and PIXalpha Dependent Activation of  **Cdc42** .  Ref: 12887923 Cell, 2003
